# National Support for Wealth-Building for Children From Low-Income Households

**DOI:** 10.1001/jamanetworkopen.2025.58092

**Published:** 2026-02-06

**Authors:** Catherine K. Ettman, Andrew Anderson, Megan V. Smith, David Radcliffe, Brian C. Castrucci, Sandro Galea

**Affiliations:** 1 Department of Health Policy and Management, Johns Hopkins Bloomberg School of Public Health, Baltimore, Maryland; 2 National Diaper Bank Network, New Haven, Connecticut; 3 Institute of Race, Power, and Politics, New School, New York, New York; 4 de Beaumont Foundation, Bethesda, Maryland; 5 Washington University in St Louis School of Public Health, St Louis, Missouri

## Abstract

This survey study examines national support for state-managed trust fund accounts for children from low-income households.

## Introduction

Although less studied than income, wealth is increasingly appreciated as a foundational driver of health.^1^ Difficulty accruing assets when starting from intergenerational disadvantage has led to interest in publicly supported wealth-building policies.^2,3^ Connecticut was the first state in the country to implement a state-wide “Baby Bonds” policy, providing $3200 in a state-managed trust fund for all babies born with Medicaid coverage, as of July 1, 2023.^4^ Depending on market performance, funds may reach $20 000 to $30 000 by early adulthood.^4^ Several other states are considering similar policies, and understanding public support may help guide future efforts.

## Methods

We estimated national support for an early wealth-building policy (ie, Baby Bonds) for low-income children using nationally representative survey data from the Cumulative Life Stressors Impact on Mental Health and Well-Being (CLIMB) Study, collected from March 27 to April 30, 2025, from the AmeriSpeak panel. Among the 2969 adults invited, 2020 completed the survey (68.0% completion rate), and 150 participants were excluded due to missing covariate data, yielding an analytic sample of 1870. Survey weights aligned the sample to the US adult population. Support for Baby Bonds was defined by a response of “strongly support” or “somewhat support” when asked, “Generally speaking, do you support or oppose creating an investment for children born into low-income households in the US”? Additional details on survey design, variable definitions, and analysis are provided in Supplement 1. This survey study followed the American Association for Public Opinion Research (AAPOR) reporting guidelines and was deemed exempt by National Opinion Research Center Institutional Review Board at the University of Chicago.

We described overall sample characteristics and assessed differences in survey-weighted prevalence of early-wealth building policy support across characteristics. We used multivariable survey-weighted logistic regression to identify factors associated with support for Baby Bonds including in the model: sex, age, race and ethnicity, household income, household savings, overall health, education, marital status, home ownership, self-reported political affiliation, and parental status. Statistical significance was set at *P* < .05, and all tests were 2-sided. Analyses were conducted in Stata 19.5 from May 23 to November 20, 2025.

## Results

The sample consisted of 1870 respondents (922 female [50.7%, weighted], 718 age 60 years or older [30.6%, weighted], 183 Black [11.5%, weighted], 286 Hispanic [17.7%, weighted], and 1,289 White [61.8%, weighted]). We found that 67.6% of US adults reported support for creating an investment account for children born into low-income households (Table). Using survey weights, Baby Bonds were supported by 541 of 662 Democrats (81.7%), 218 of 492 Republicans (48.0%), 314 of 494 Independents (67.5%), and 142 of 222 of other adults (69.0%) (Figure). In the adjusted regression model, having no or low savings ($0 or $1 to $20 000 relative to $200 000 or more) and identifying as Democrat, Independent, or none of the above (relative to Republican) were the only variables significantly associated with support for Baby Bonds.

**Table.** Baby Bonds Support Among US Adults by Characteristics

Characteristics^a^	Total respondents, No. (weighted %)	Respondents, No. (%)^b^		*P* value^c^
	Total	Oppose	Support	
Total	1870 (100.0)	655 (32.4)	1215 (67.6)	
Sex				
Male	948 (49.3)	332 (32.9)	616 (67.1)	.71
Female	922 (50.7)	323 (31.9)	599 (68.1)	
Age, y				
18-29	134 (20.0)	36 (24.4)	98 (75.6)	<.001
30-44	567 (25.2)	144 (23.5)	423 (76.5)	
45-59	451 (24.2)	178 (39.0)	273 (61.0)	
≥60	718 (30.6)	297 (39.8)	421 (60.2)	
Race and ethnicity				
Black	183 (11.5)	42 (22.0)	141 (78.0)	<.001
Hispanic	286 (17.7)	74 (23.4)	212 (76.6)	
White	1289 (61.8)	504 (37.5)	785 (62.5)	
Other	112 (9.1)	35 (28.9)	77 (71.1)	
Household income, $				
0 to <45 000	525 (34.0)	147 (24.9)	378 (75.1)	.002
45 000 to <75 000	412 (20.6)	158 (35.7)	254 (64.3)	
75 000 to <150 000	633 (29.8)	241 (36.9)	392 (63.1)	
≥150 000	300 (15.6)	109 (36.0)	191 (64.0)	
Household savings, $				
0	268 (17.4)	74 (25.3)	194 (74.7)	<.001
1 to <20 000	531 (31.7)	146 (22.3)	385 (77.7)	
20 000 to <200 000	548 (27.2)	222 (41.0)	326 (59.0)	
≥200 000	523 (23.7)	213 (41.4)	310 (58.6)	
Household debt, $				
0	469 (26.3)	212 (40.9)	257 (59.1)	.01
1 to <5000	465 (26.0)	158 (28.5)	307 (71.5)	
5000 to <25 000	485 (26.4)	151 (30.5)	334 (69.5)	
≥25 000	446 (21.3)	133 (29.2)	313 (70.8)	
Self-reported overall health				
Fair or poor	404 (21.8)	122 (29.7)	282 (70.3)	.35
Good or above	1466 (78.2)	533 (33.2)	933 (66.8)	
Education				
Some college or less	1133 (63.8)	411 (31.9)	722 (68.1)	.58
College or more	737 (36.2)	244 (33.4)	493 (66.6)	
Marital status				
Not married	836 (52.4)	258 (28.0)	578 (72.0)	<.001
Married or living with partner	1034 (47.6)	397 (37.2)	637 (62.8)	
Religious service attendance				
Never attended	641 (33.6)	206 (29.1)	435 (70.9)	.08
Ever attended	1229 (66.4)	449 (34.1)	780 (65.9)	
Employment status^d^				
Not employed	64 (5.1)	13 (15.6)	51 (84.4)	.005
Employed or other	1805 (94.9)	641 (33.3)	1164 (66.7)	
Home ownership^d^				
Does not own home	601 (36.5)	157 (23.7)	444 (76.3)	<.001
Owns home	1269 (63.5)	498 (37.4)	771 (62.6)	
Political affiliation				
Democrat	662 (34.6)	121 (18.3)	541 (81.7)	<.001
Republican	492 (25.8)	274 (52.0)	218 (48.0)	
Independent	494 (26.4)	180 (32.5)	314 (67.5)	
None of the above	222 (13.2)	80 (31.0)	142 (69.0)	
Parental status				
Minor and adult	198 (11.2)	65 (34.4)	133 (65.6)	<.001
Minor only	347 (16.9)	91 (22.8)	256 (77.2)	
Adult only	704 (32.5)	297 (41.1)	407 (58.9)	
No children	621 (39.4)	202 (28.9)	419 (71.1)	

^a^ Cumulative Life Stressors Impact on Mental Health and Well-Being (ie, CLIMB) Wave 6 Data were from March to April 2025.

^b^ Oppose and support columns show row percentages. Total column shows column percentages.

^c^ Tests of association estimated using Pearson χ^2^ tests for categorical variables.

^d^ Missing values: household debt, n = 5; employment, n = 1.

**Figure.**
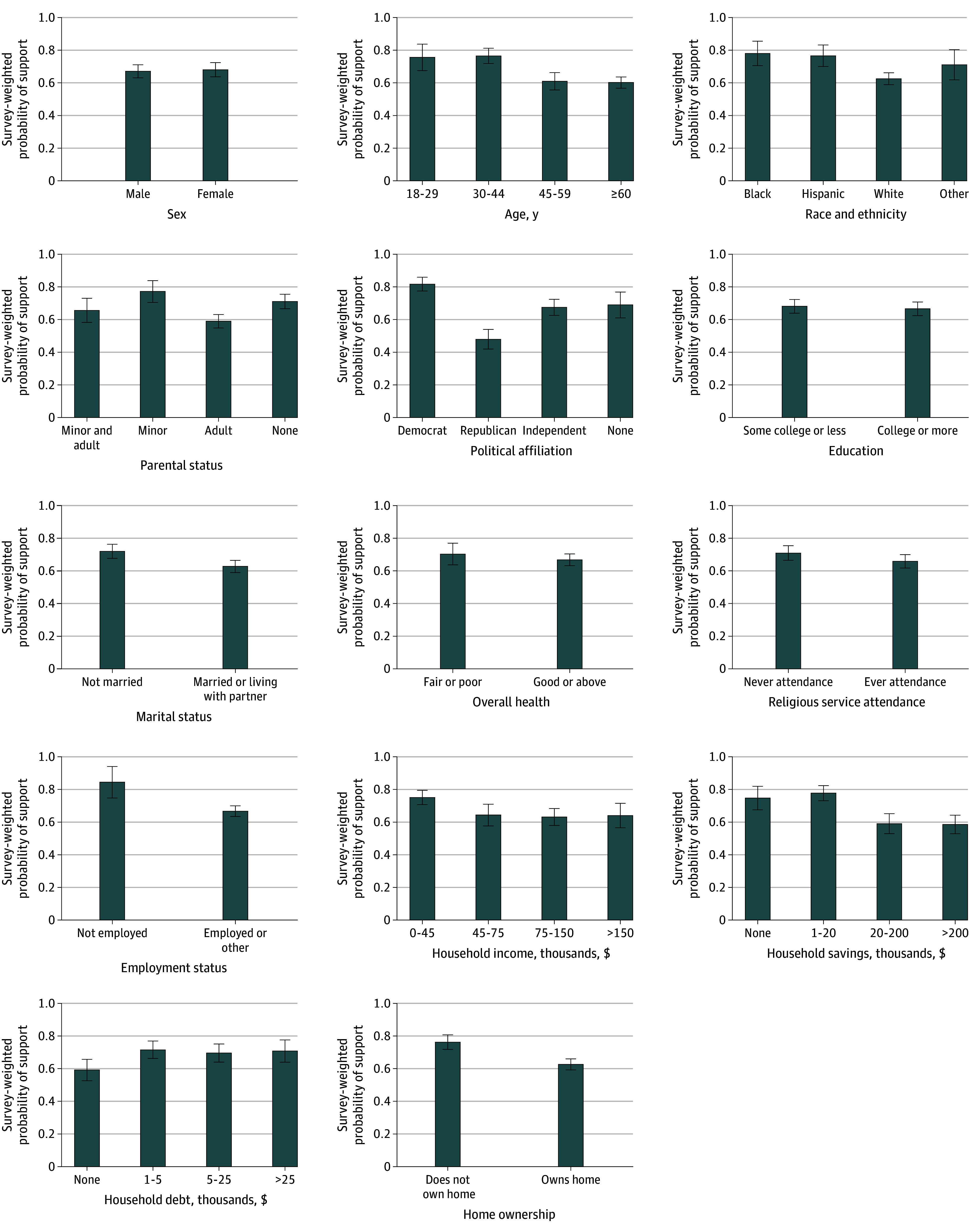
Support for Baby Bonds by Demographic Characteristics and Assets Cumulative Life Stressors Impact on Mental Health and Well-Being (ie, CLIMB) Wave 6 data used, collected March to April 2025 (n = 1870). Survey weights applied.

## Discussion

Most US adults supported wealth-building policies for children from low-income families, such as Baby Bonds. Almost half of Republicans and more than two-thirds of all other political groups (Democrat, Independent, and None) supported Baby Bonds. These findings provided an updated national perspective on support for Baby Bonds programs,^4^ building on earlier work by the American Civil Liberties Union in December 2022^2^ finding that 61% of voters supported Baby Bonds.^2^

This study has limitations. The survey did not assess preferences for different level of investments through Baby Bonds, limiting inference about other programs that provide financial support to children.

The creation of Trump Accounts through the 2025 Budget Reconciliation Act suggests a window of opportunity to advance child-oriented wealth-building programs. Trump Accounts provide $1000 for babies born between 2025 and 2028.^5^ Unlike Baby Bonds, which are automatic and income-targeted, Trump Accounts allow voluntary contributions that may be less feasible for lower-resourced families.^6^ Nonetheless, the combined momentum behind Baby Bonds and Trump Accounts may create new opportunities to advance early-life wealth building policies that promote population health.

## Notes

Supplement 1.
eMethods.

Supplement 2.
Data Sharing Statement
