# Supplementary material for: National Support for Wealth-Building for Children From Low-Income Households
Source: JAMA Netw Open. 2026 Feb 6;9(2):e2558092. doi: 10.1001/jamanetworkopen.2025.58092 (PMC12881980; doi:10.1001/jamanetworkopen.2025.58092)
Supplement: Supplement 1. — eMethods. [file jamanetwopen-e2558092-s001.pdf]

Supplemental Online Content

Ettman CK, Anderson A, Smith MV, Radcliffe D, Castrucci BC, Galea S. National support for wealth-building for children from low-income households. *JAMA Netw Open*. 2026. 9(2):e2558092. doi:10.1001/jamanetworkopen.2025.58092

eMethods.

This supplemental material has been provided by the authors to give readers additional information about their work.

© 2026 Ettman CK et al. *JAMA Network Open*.

eMethods. Survey design

The Cumulative Life Stressors Impact on Mental Health and Well-Being (CLIMB) Study is a longitudinal panel measuring population trends, assets, and policy preferences in U.S. adults. Run annually in March-April since 2020, the CLIMB Study includes U.S. adults recruited from the AmeriSpeak panel, fielded by National Opinion Research Center at the University of Chicago. The AmeriSpeak panel uses a multi-stage recruitment strategy based on a national sample frame that represents 97% of U.S. households. Invited participants are contacted by mail, by phone, and by in-person interviewers. U.S. adults who are not included in the sampling frame include people who only have P.O. box addresses, newly constructed dwellings, and some addresses that are not in the USPHS Delivery sequence file. The CLIMB Survey was delivered in English. Participants could take the CLIMB survey over the internet or by phone. Consent was provided in writing to participate in the AmeriSpeak panel and at the start of each wave of the CLIMB survey. The parent study for data collection was deemed exempt by the IRB at the NORC at the University of Chicago. The secondary analysis of deidentified survey data was deemed Not Human Subjects Research by the Johns Hopkins University School of Public Health IRB. Wave 6 of the CLIMB study was fielded from March 27 to April 30, 2025.

Variable definitions

Support for Baby Bonds was defined as a binary variable (combining “somewhat support” or “strongly support”) in response to the following question: “Recently, some have proposed a new policy which would create an investment for each newborn child born into poverty. The child would be eligible to receive the investment as an adult. The value of the investment would start at about $3,000 when the child is born and could be worth up to about $20,000 when the child reaches adulthood. Recipients can access these funds starting at 18, but must use them towards wealth building activities like purchasing a home, starting or buying a business, starting a retirement fund, or education such as a college degree or vocational training. Generally speaking, do you support or oppose creating an investment for children born into low-income households in the U.S.?” Answer options included: strongly support, somewhat support, somewhat oppose, strongly oppose.

Demographic variables included age quartiles (“18-29”, “30-44”, “45-59”, “≥60)”, respondent sex (“male”, “female”), and self-reported mutually exclusive race and ethnicity categories (“White”, “Black”, “Hispanic”, “Other”).

Assets included financial assets (household income, savings, debt), physical assets (home ownership), social assets (educational, marital status, employment, religious service attendance) based on the Asset Framework to Guide Nonhealth Policy for Population Health[1],

Financial asset variables included quartiles of household income (“$0 to <$45,000”, “$45,000 to <$75,000”, “$75,000 to <$150,000”, “≥$150,000”), household savings (“No savings”, “$1 to < $20,000”, “$20,000 to <$200,000”, “≥$200,000”), and household debt (“No debt”, “$1-$5,000”, “$5,000-$250,000”, and “≥$25, 000”).

© 2026 Ettman CK et al. *JAMA Network Open*.

Physical assets included home ownership (versus renting or other). Home ownership was defined as “owns home” or “does not own home” (combining responses of “I rent the place where I live”, or “I occupy the place where I live without payment of cash rent”). 7 participants were missing home ownership responses in 2025; missing data were imputed based on respondent answers to the same question in the CLIMB Study Wave 5 (5 recovered), Wave 4 (1 recovered) and Wave 3 (1 recovered).

Social assets included education (“Some college or less”, “College or above”); marital status (“not married”, “married or living with partner”); employment, and religious service attendance. Employment was assessed using the following question: “Which of these options best describes your current work situation?” Participants were able to choose one of the following responses: “Working full-time”, “Working part-time”, “Looking for work or unemployed”, “Retired”, “A homemaker”, “A student”, “On maternity or paternity leave”, “On illness or sick leave”, “On disability”, or “Other – please specify” (text response). Employment was then operationalized as a binary variable using “Unemployed or looking for work” or “Employed or other” (defined as any response other than “looking for work or unemployed”) based on previously published work.[2] Religious service attendance was assessed using the following question: “How often do you attend religious services?”. Responses were combined into a binary variable using “Never attendance” (defined by a response of “Never”) and “Ever attendance” (defined as any other response, ranging from “several times a week” to “once a year”).

Self-reported overall health was defined as “Good or above” overall health (including “Excellent”, “Very good”, and “Good”) or “Fair or poor” overall health (including “Fair” and “Poor”) in response to the question: “In general, how would you rate your overall health?”

Political affiliation was assessed using the following question: “Do you consider yourself a Democrat, a Republican, an Independent or none of these?”. Participants were able to choose one of the following responses: “Democrat”, “Republican”, “Independent”, or “None of these”.

Parental status was defined as having both “minor and adult children”, “minor children only”, “adult children only”, and “no children”. This measure was derived from two survey questions with yes/no response choices: 1) “Are you the parent, guardian, or primary caregiver of any children under 18 years old?”, and 2) “Are you the parent of any children over 18 years old?”. The variable was defined using four levels based on the responses to each question: “Minor and adult children” for those who responded affirmatively to both, “Minor children only” and “Adult children only” for those who responded affirmatively to only one, and “No children” for those who responded “No” to both.

1. Ettman CK, Galea S. An Asset Framework to Guide Nonhealth Policy for Population Health. JAMA Health Forum 2024; 5: e241485–e241485.

2. Hatton CR, Ettman CK, Gollust S, Abdalla SM, Galea S. Mental health and U.S. attitudes towards social determinants of health policies. American Journal of Preventive Medicine 2024; 0. DOI:10.1016/j.amepre.2024.05.015.

© 2026 Ettman CK et al. *JAMA Network Open*.

Analysis

First, we describe the CLIMB Wave 6 sample. Among the 2,969 adults invited to take Wave 6 of the CLIMB Study, 2,020 completed the survey (68.0% completion rate). Among those, n=1,870 had no missing values for sex, age, race, household income, household savings, self-reported overall health, education, marital status, home ownership, self-reported political affiliation, and parental status.

We present unweighted frequencies and weighted percentages of characteristics of the included sample. Survey weights were provided by NORC for qualified completed responses for Wave 6 and weighted to the U.S. population benchmarked to March 2024 Current Population Survey (CPS) data.

Second, we estimated the odds ratio of support for Baby Bonds using a survey-weighted logistic regression model. The regression model included: sex, age, race and ethnicity, household income, household savings, overall health, education, marital status, home ownership, self-reported political affiliation, and parental status. Religious service attendance, employment, and household debt were not included in regression models. The model incorporated the specific survey design, such as the primary sampling units and strata, using the Stata svy command and subpop() command to estimate variance in relation to the full weighted population.

© 2026 Ettman CK et al. *JAMA Network Open*.
