# Supplementary material for: National Support for Wealth-Building for Children From Low-Income Households
Source: JAMA Netw Open. 2026 Feb 6;9(2):e2558092. doi: 10.1001/jamanetworkopen.2025.58092 (PMC12881980; doi:10.1001/jamanetworkopen.2025.58092)
Supplement: Supplement 2. — Data Sharing Statement [file jamanetwopen-e2558092-s002.pdf]

Data Sharing Statement

Data
Data available: Yes
Data types: Data dictionary
How to access data: Data available for research purposes with reasonable request to cettman1@jhu.edu.
When available: With publication

Supporting Documents
Document types: Statistical/analytic code
How to access documents: Data available for research purposes with reasonable request to cettman1@jhu.edu.
When available: With publication

Additional Information
Who can access the data: Data available for research purposes with reasonable request to cettman1@jhu.edu.
Types of analyses: Data available for research purposes with reasonable request to cettman1@jhu.edu.
Mechanisms of data availability: Data available for research purposes with reasonable request to cettman1@jhu.edu.
